# Clinical Correlation of Placental Pathology in Medical Disorders of Pregnancy and Its Comparison in Normal Pregnancy

**DOI:** 10.7759/cureus.111359

**Published:** 2026-06-23

**Authors:** Prashant Vasnani, Pragati Awasthi, Deepshikha Verma

**Affiliations:** 1 Department of General Medicine, Jawaharlal Nehru (JLN) Medical College and Hospital, Ajmer, IND; 2 Department of Pathology, Atal Bihari Vajpayee Government Medical College, Vidisha, IND

**Keywords:** eclampsia, gdm (gestational diabetes mellitus), pih (pregnancy-induced hypertension), placental insufficiency., preeclampsia

## Abstract

Introduction

The placenta serves as the vital interface between mother and fetus, reflecting maternal and fetal health. Pregnancy-related disorders such as gestational diabetes, pregnancy-induced hypertension (PIH), preeclampsia, and eclampsia can cause structural and functional placental alterations, leading to adverse perinatal outcomes.

Methods

A prospective observational study was conducted over two months in the Departments of Pathology and Obstetrics and Gynecology. Thirty-seven placentas from 36 pregnancies were examined following informed consent. Gross morphology and histopathological features were evaluated using hematoxylin and eosin (H&E) staining and correlated with maternal clinical data.

Results

Mean placental weight was highest in diabetic pregnancies (716 g) and lowest in PIH and preterm premature rupture of membranes (PPROM) (450 g). The most frequent histopathological findings included increased syncytial knots (86.4%), fibrinoid necrosis (81%), hemorrhage (78.3%), chorangiosis (75.6%), and calcification (67.5%). Medial vessel wall thickening, avascular villi, and calcification were primarily associated with hypertensive disorders, indicating uteroplacental insufficiency. Similar but less severe lesions, such as hemorrhage and fibrinoid necrosis, were also noted in normal placentas.

Conclusion

Common placental lesions, particularly syncytial knot formation, fibrinoid necrosis, and chorangiosis, were observed across both normal and complicated pregnancies, with higher frequency and severity in maternal disorders. These findings underscore the placenta’s diagnostic value as a sensitive indicator of maternal vascular and hypoxic changes, emphasizing its importance in understanding pregnancy complications and fetal outcomes.

## Introduction

The placenta forms a connection between the fetus and the uterine wall of the mother. It attaches to the wall of the uterus, usually the top, side, front, or back of it. It plays an important role in the excretory, hepatic, gastrointestinal, endocrine, respiratory, and immune systems [1]. The umbilical cord arises from it and forms a conduit between the fetus and the placenta. It is indicative of the prenatal life of the baby.

Placental health can be affected by various factors, including maternal age, premature rupture of membranes, blood pressure, abdominal trauma, substance abuse, twins or multiple pregnancy, blood clotting disorders, various infections, placental complications, etc. Placental dysfunction is implicated in many major complications of pregnancy, which can lead to adverse maternal and infant outcomes, such as preeclampsia, fetal growth restriction, and stillbirth [2]. Thus, careful examination of the placenta in many pregnancy-related medical disorders can help us understand their etiology. Disorders like gestational diabetes, pregnancy-induced hypertension (PIH), eclampsia, and preeclampsia result in destruction of the placenta and alteration in its functions, causing placental insufficiency [3]. A careful examination of the placenta, along with a microscopic study, may frequently shed light on many causes of perinatal death. Nowadays, when medicolegal problems threaten practicing obstetricians, a study of the placenta with histology can prove the exact pathology behind perinatal death and act as a legal shield for the doctor from the consumer forum [4].

PIH can cause distress and fatality in the mother, fetus, and newborn [5]. Fetuses in these conditions are most likely to suffer from intrauterine growth retardation, prematurity, and intrauterine death. Preeclampsia is a hypertensive condition to the extent of 140/90 mmHg, along with edema and proteinuria. It occurs after the 20th week of gestation. Eclampsia is a condition in which preeclampsia is accompanied by the onset of convulsions. Hypertension, eclampsia, preeclampsia, and intrauterine growth restriction (IUGR) can lead to preterm labor [6]. Gestational diabetes mellitus (GDM) is a condition in which the blood sugar level is increased, and it occurs in the second part of the pregnancy. Most women do not have hyperglycemia after delivery [7]. Babies born to GDM mothers suffer from fetal macrosomia (birth weight >4000 g) [8].

Preeclampsia (PE) and fetal growth restriction (FGR) are associated with an increased risk of maternal and perinatal morbidity, as well as mortality. FGR is estimated to be the underlying cause in 30 to 50% of cases of stillbirths [9,10]. Placental studies like these and screening of placental abnormalities during the antenatal period with the help of sonography can help us in the management of several conditions like the ones mentioned above. By comparing the placenta associated with medical disorders of pregnancy with placentas of normal pregnancy, we can have a better understanding of the pathology of such disorders and the factors responsible for them. It can help us with the management of subsequent pregnancies and prevent their tendency to recur, and can be helpful in planning the future care for the mother and child.

Aims

The present study aimed to study gross and pathological (morphology and histology) of the placenta in medical disorders of pregnancy, to establish a possible correlation between placental abnormalities and the outcome of pregnancy, and to study changes specific to some particular medical disorders, if present. 

## Materials and methods

The present study was a hospital-based prospective study that was carried out from July 1 to September 2, 2018, at the Department of Pathology in collaboration with the Department of Obstetrics and Gynecology at our college-associated hospital over the span of a given time period (2 months). Placentas were collected just after the delivery, comprising placentas associated with medical disorders of pregnancy and the normal placentas. A total of 37 placentas from 36 pregnancies were collected for this study. Detailed obstetric and medical histories were recorded. The entire project was done with the consent of patients and after getting ethical clearance from our college's ethical committee. The placentas with attached membranes and umbilical cords were collected soon after delivery, washed in phosphate buffer saline to remove blood contamination, labelled, and then fixed with 10% buffered formalin overnight. Blood clots, if present, were removed. Gross examination of placenta included length, width, thickness in cm, weight, shape, length and diameter of umbilical cord, insertion, twists, knots in umbilical cord, color, hematomas in membranes, number of vessels and presence of masses, thrombi, fibrin, infarction at fetal and maternal surface, calcification, infarction, strictures, ulcers, hyper/hypocoiling, present of masses, etc.

Sections were taken from the umbilical cord, membrane, and maternal and fetal surfaces of the placenta. For microscopic examination, placental tissue was processed, and 4 µm thick sections were obtained for histopathological examination. Rehydration with an ethanol series and staining with hematoxylin and eosin (H&E) were done. In each placental slide, the 10 smallest terminal villi were observed in 10 different fields (magnification × 400) under light microscopy. Microscopic examination included examination of the following: trophoblast abnormalities, stromal abnormalities, villous vessel abnormalities, calcification, and other abnormalities

Morphological and histological changes of the placenta in medical disorders of pregnancy were studied and compared with the normal placentas, and a correlation between placental abnormality and pregnancy outcome was established wherever possible.

Statistical analysis

Data were entered and analyzed using MS Excel (Microsoft Corporation, Redmond, Washington, United States) and IBM SPSS Statistics for Windows, Version 22 (Released 2013; IBM Corp., Armonk, New York, United States). Descriptive statistics, including mean, standard deviation, and percentage distribution, were used to summarize continuous and categorical variables. The mean values of placental and fetal weights were compared among groups using the Student’s t-test and one-way analysis of variance (ANOVA) wherever applicable. A p-value of <0.05 was considered statistically significant.

## Results

In the given time period of two months (July 1 to September 1, 2018), 37 placentas were collected from 36 pregnancies, including an additional placenta from a diamniotic dichorionic twin pregnancy. The age range of mothers was between 20 and 37 years. Placentas were collected from our college-associated hospital, comprising 11 placentas from normal pregnancies (29.7%), seven placentas of anemia cases (18.9%), three placentas of PIH cases (8.1%), four placentas of hypothyroid cases (10.8%), three placentas of urinary tract infection (UTI) cases (8.1%), three placentas of diabetic cases (8.1%), two placentas of preterm premature rupture of membrane (PPROM) cases (5.4%), and two placentas of eclamptic and preeclamptic cases (5.4%). In some of the cases, these clinical conditions were found to coexist. A placenta of an intrauterine fetal death (IUFD) baby was also collected, along with the placenta of an aborted fetus. Two cases of twin pregnancy (5.5%) were found among these, one being dichorionic diamniotic and the other being monochorionic and monoamniotic. On the basis of gestational duration, out of 33 pregnancies resulting in 34 live birth placentas, the number of preterm placentas was 12 (36.36%), the number of term placentas was 21 (63.63%), and a single placenta (3%) was from a post-term pregnancy. Among the total placentas, four belonged to IUGR babies (10.8%). Variations in placental and fetal weight were observed throughout the study.

The mean placental weight was the greatest for post-term babies (600 g) and the least for IUGR babies (550 g), though a placenta weighing 350 g was also collected, belonging to an IUFD at 24 weeks and six days. The mean fetal weight was the greatest for full-term babies (2.78 kg) and the least for IUGR babies (1.80 kg). The mean fetal-to-placental ratio was the greatest for term babies (4.96) and the least for IUGR babies (3.27) (Table 1).

**Table 1:** Mean placental and fetal parameters with relation to fetal growth pattern IUGR: intrauterine growth restriction; IUFD: intrauterine fetal death

Fetal growth	Maternal age	Mean fetal	Mean placental	Mean F:P
Full term (N = 21)	20-37 yrs	2.78 kg	560.47 g	4.96
Preterm (N = 12)	20-28 yrs	2.16 kg	575 g	3.75
IUGR (N = 4)	20-23 yrs	1.80 kg	550 g	3.27
Post-term (N = 1)	32 yrs	2.70 kg	600 g	4.50
IUFD (N = 1)	30 yrs	-	350 g	-

Among various diseases, placentas with the greatest mean placental weight belonged to diabetes (716.66 g), and placentas with the least mean placental weight belonged to PPROM and PIH (450 g each). The mean fetal weight was greatest in diabetes (2.7 kg) and least in eclampsia/preeclampsia (2.25 kg). The fetal-to-placental ratio was greatest in PPROM and least in diabetes (Table 2).

**Table 2:** Mean placental and fetal parameters with relation to different diseases PPROM: preterm premature rupture of membrane; GDM: gestational diabetes mellitus; DM: diabetes mellitus; PIH: pregnancy-induced hypertension; UTI: urinary tract infection; F:P ratio: fetal-to-placental ratio

Risk factors	Maternal age range	Mean fetal weight	Mean placental weight	Mean F:P ratio
PPROM N = 2	25-32 yrs	2.5 kg	450 g	5.55
Anemia N = 7	20-33 yrs	2.51 kg	585.71 g	4.28
GDM/DM N = 3	26-30 yrs	2.7 kg	716.66 g	3.76
PIH N = 3	22-26 yrs	2.36 kg	450 g	5.24
Eclampsia/preeclampsia N = 2	22-27 yrs	2.25 kg	575 g	3.91
Hypothyroidism N = 4	21-33 yrs	2.67 kg	575 g	4.64
UTI N = 3	23-33 yrs	2.58 kg	550 g	4.69
Normal N = 11	21-37 yrs	2.61 kg	551.81 g	4.72

The number of round-shaped placentas was 23 (62%), and oval-shaped, 12 (32.4%). Two (5.4%) placentas with irregular shapes were also collected. Placentas with eccentric insertion were 23 (62.1%) in number, and those with central insertion were 14 (37.8%). All the placentas had marginal insertions of membranes. The mean placental size was 15.5 cm X 13.8 cm, and the mean placental thickness was 2.95 cm. The mean umbilical cord length was 24.2 cm. All umbilical cord specimens had two arteries and one vein, with one exception having one artery and one vein, which was associated with fetal distress and oligohydramnios. No prominent number of knots was observed in umbilical cords. Hemorrhage on the maternal surface was present in 37.8% of cases along with clots, and calcification was present in 27% of cases. Complete infarction on the maternal and fetal surface was seen in a placenta belonging to the case of intrauterine fetal death. Hemorrhage was not frequently seen on the fetal surface, but calcification on the fetal surface was present in 13.5% of cases. Hematoma of membranes was also seen in 16.2% of cases.

Histological analyses

An increase in syncytial knots was the most dominant finding in the study, comprising 86.4% of the placentas, and it was found in the maximum number in the diabetics, PIH, eclampsia/preeclampsia, and UTI groups. Fibrinoid necrosis in 81% and hemorrhage in 78.3% were also found frequently and were maximum in cases with hypothyroidism, PIH, eclampsia/preeclampsia, anemia, and UTI. Chorangiosis was found in 75.6% of cases, occurring most often in cases of PPROM and UTI. Calcification was found in 67.5% of cases, occurring most often in cases of PIH and preeclampsia/eclampsia. Immature and avascular villi were seen in 27% of cases, mostly seen in cases of PIH and eclampsia/preeclampsia. Medial vessel wall thickening was found in 27% of cases, and like calcification and avascular villi, they were mostly associated with PIH and preeclampsia/eclampsia. Decidualization was found in 35.1% of cases and was mostly seen in diabetics. Chorioamnionitis was found in 16.2% of cases and occurred mostly in cases of PPROM. The least observed histopathology was acute funisitis, seen in 6.25% and only seen in 50% of cases of PPROM. The most common findings in normal placentas were hemorrhage (90%) and increased syncytial knots (81.80%) (Table 3).

**Table 3:** Histopathological findings in different diseases PIH: pregnancy-induced hypertension; PPROM: preterm premature rupture of membranes; UTI: urinary tract infection; GDM: gestational diabetes mellitus; DM: diabetes mellitus

Histopathological feature	Hypothyroidism (n = 4)	PIH (n = 3)	Eclampsia/preeclampsia (n = 2)	PPROM (n = 2)	Anemia (n = 7)	UTI (n = 3)	GDM/DM (n = 3)	Normal (n = 11)
Syncytial knots	75%	100%	100%	50%	85.7%	100%	100%	81.8%
Chorioamnionitis	25%	-	-	50%	14.2%	-	-	18.2%
Chorangiosis	75%	66.6%	50%	100%	57%	100%	66.6%	54.5%
Calcification	75%	100%	100%	50%	57%	33.3%	33.3%	63.6%
Medial vessel wall thickening	-	100%	100%	-	-	-	-	-
Avascular or immature villi	25%	66.6%	50%	-	28.5%	33.3%	-	27.3%
Fibrinoid necrosis	100%	100%	100%	50%	85.7%	66.6%	66.6%	63.6%
Hemorrhage	100%	66.6%	50%	50%	71.4%	100%	66.6%	90.0%
Decidualization	25%	33.3%	-	50%	42.8%	33.3%	66.6%	45.4%
Funisitis	-	-	-	50%	-	-	-	-

The most common histopathology seen in preterm cases was calcification, followed by fibrinoid necrosis, hemorrhage, and an increase in syncytial knots. The most common histopathology in term babies was an increase in syncytial knots followed by hemorrhage and fibrinoid necrosis, whereas in IUGR cases, the most common histopathology was calcification, avascular and immature villi, and an increase in syncytial knots along with hemorrhage. Placenta from post-term delivery had fibrinoid necrosis, calcification, an increase in syncytial knots, an increase in medial vessel wall thickening, and hemorrhage. The placenta of IUFD had congestion, excessive fibrinoid necrosis, an increase in syncytial knots, and hemorrhage. Some of these findings were also present in placentas from normal cases with no clinical conditions like fibrinoid necrosis, hemorrhage, increased syncytial knots, or calcification (Table 4).

**Table 4:** Histopathological findings in different fetal growth patterns IUGR: intrauterine growth restriction

Histopathological feature	Preterm (n = 12)	Term (n = 21)	Post-term (n = 1)	IUGR (n = 4)
Syncytial knots	83.3%	80.9%	100%	100%
Chorioamnionitis	8.3%	19.0%	-	-
Chorangiosis	58.3%	62.0%	-	75.0%
Calcification	91.6%	52.3%	100%	100%
Medial vessel wall thickening	25.0%	9.5%	100%	50.0%
Avascular or immature villi	66.6%	23.8%	-	100%
Fibrinoid necrosis	83.3%	71.4%	100%	75.0%
Hemorrhage	83.3%	76.0%	100%	100%
Decidualization	58.3%	28.5%	-	50.0%
Funisitis	-	4.7%	-	-

## Discussion

In our study, the mean placental weight of full-term (560.47 g), preterm (575 g), PPROM (450 g), anemia (585.71 g), diabetes (716.66 g), and preeclampsia (575 g) was found to be greater than that observed in Ramachandra et al. The mean placental weights of full-term, preterm, PPROM, anemia, diabetes, and preeclampsia in Ramachandra et al. were 425.52 g, 316.9 g, 418 g, 406 g, 375 g, and 404.7 g, respectively [4].

In hypothyroidism, the mean placental weight was 575 g; in the findings of Ozogul et al., the mean placental weight was slightly more (587.5 g) [9]. In PIH, the mean placental weight was 450 g. In the findings of Ahmed et al., the mean placental weight was less (382.25 g) [11]. Funisitis is recognized as the histologic manifestation of the fetal inflammatory response and has been associated with adverse neurological outcomes [12].

The mean placental weight and thickness of a normal full-term placenta were 551.81 g and 2.95 cm, respectively. In the findings of the term placenta of Ramachandra et al., the mean placental weight (508 g) and thickness (2.3 cm) were lower [4]. These discrepancies in mean values in this study and other studies must be due to regional differences.

Irregular nonovoid shapes are the result of abnormal implantation, failed involution of chorionic villi, and intracavitary uterine abnormalities [4]. In this study, the majority of placentas were round-shaped (62%). Oval-shaped placentas were 32.4%, and 5.6% were irregularly shaped, showing similarities with the findings of Ramachandra et al., where round-shaped placentas were in the majority (74.16%), oval-shaped were 17.5%, and bilobed were 5.83% [4]. An increased number of syncytial knots, avascular and hypovascular villi, and a large amount of fibrinoid necrosis, suggesting infarction, were found in the case of anemia. Similar findings were also found in Biswas et al. These findings are suggestive of hypoxia and reduced placental perfusion [13]. Increased numbers of syncytial knots were also seen in hypothyroid cases, which was a consistent finding in Ozogul et al. Along with syncytial knotting, chorangiosis and calcification were also seen prominently in hypothyroid cases. An increase in syncytial knots indicates alterations in hormonal factors, which may lead to a change in placental morphology [9].

All the placentas had normal umbilical cord insertion; among them, 62.1% were eccentric and 37.8% had central insertion. No velamentous, marginal, or furcate insertion of cords was found. Similar findings were reported in Shrivastava et al. [14], where the number of placentas having eccentric insertion was greater than that of those having central insertion. Along with these, they also reported furcate and marginal insertion of placentas in their study. Such variation in the study must be due to trophotrophism [15]. No significant pattern of umbilical cord insertion and pregnancy complications was found in this study. All the umbilical cords consisted of two arteries and one vein, except for a case of fetal distress and oligohydramnios in which the umbilical cord consisted of only one artery and vein. The incidence of a single umbilical artery is 0.5-1%, according to Geipel et al., and it arises due to primary atrophy and can be associated with congenital anomalies [16].

A placenta of IUFD had diffuse infarction on the maternal and fetal surfaces, which is suggestive of uteroplacental insufficiency. There can be several causes of uteroplacental insufficiency, including hypertensive diseases, anemia, drug abuse, blood clotting disorders, diabetes, etc. According to Dash et al., 10.26% of IUFD had infarction as the main pathological change in placentas [17].

Histological analysis

Avascular and immature villi and decidualization were found more dominantly in preterm as compared to term and post-term. A study by Schweikhart et al. observed 33% of preterm placentas having an increased number of synchronous villous patterns [18]. An increase in immature villi can be seen in diabetes and preeclampsia, also. A few studies also propose that in preterm placentas, villous immaturity is difficult to assess and reliably recognize. The cause of failure of villous maturation is not known; it can’t be simply a reflection of a failure of fetal maturation. Infrequent evidence of it in preeclamptic placentas suggests that uteroplacental ischemia is not a significant etiologic factor. Chorangiosis was seen most in IUGR placentas. Similarities in the findings of Gupta et al. and their association with preeclampsia suggest that placental tissue hypoxia causes callous capillary endothelial proliferation [19].

When analyzing placentas from various diseases of pregnancy, it was found that medial vessel wall thickening and immature villi had the most association with PIH, eclampsia, and preeclampsia cases. The results were in accordance with the study by Salmani et al. Immature villi and medial cell wall proliferation are the result of hypertensive conditions and compromised blood supply. Maternal uteroplacental blood flow decreases due to maternal vasospasm, which may lead to further constriction of fetal stem arteries [10]. Cases of diabetes were mostly associated with increased numbers of syncytial knots and chorangiosis. Similar observations were done in Augustine et al. Excessive syncytial knotting and chorangiosis are indicators of excessive chorionic villous capillary proliferation and exaggerated senescence induced by fetal hypoxia [20]. Findings of Li et al. indicate that gestational diabetes induces excessive chronic hypoxia stress and inflammatory response in placentas, which may contribute to the high risks of perinatal complications of obesity and diabetic mothers [21].

Chorioamnionitis and acute funisitis were found in the case of PPROM, which was found consistent with the findings of Armstrong-Wells et al. Both of these findings suggest an inflammatory pathology of the rupture of membranes. Funisitis is also seen as an indicator of fetal-derived inflammation and has been associated with adverse neurological outcomes. Chorioamnionitis is seen less prevalently with later gestational age, indicating that women who develop placental inflammation may go into labor sooner than those who haven’t [12]. Placentas from cases of UTIs had many nonspecific findings, including increased syncytial knotting and chorangiosis. Nothing much can be said about the placental changes caused by UTI. According to Ozer et al., acute chorioamnionitis, chronic villitis, and decidualization were frequently found in the placenta of UTI cases [22]. Fibrinoid necrosis, hemorrhage, and calcification were frequent findings in placentas from normal cases and with other disorders. These may be a result of other complications like fetal distress, infections, and several other idiopathic conditions.

Limitations

The study was limited by its small sample size and short duration, restricting the statistical power and generalizability of findings. Some patients had coexisting medical disorders, which may have confounded the correlation between specific placental changes and individual conditions. Additionally, quantitative morphometric analysis and immunohistochemical correlation were not performed, which could have provided deeper insights into the pathophysiological mechanisms involved.

## Conclusions

This study aimed to find pathologic changes in placentas of different pregnancy-related diseases and their clinical correlation. It was observed that there was an increase in chorangiosis and syncytial knots wherever uteroplacental hypoperfusion was involved, suggesting hypoxia as the basis of the pathology of diseases. Hemorrhage, fibrinoid necrosis, and calcification were found commonly in almost all types of placentas. Specific pathologic changes could not be observed, as in many cases, diseases were found to be coexisting, and the sample size was limited. Similarities in the pathology of various diseases were noticed. Fetal and placental weight were found to be increased in diabetes and decreased in IUGR. Vessel wall thickening was found in each case of IUGR, indicating malperfusion and hypertensive pathology behind the placental insufficiency, causing restriction in the growth of the fetus. Chorangiosis was found as an adaptive measure to overcome the lack of fetal oxygenation. Pathologies of different diseases were found to be interrelated in the majority of cases, suggesting a primary basis of morphological and physiological changes that can later lead to different manifestations and can affect fetal outcome in adverse ways. As the placenta is an easily accessible organ, its gross and histopathological study must be done in case of adverse outcomes of pregnancy, which can help us in better management of diseases having a recurrent tendency in subsequent pregnancies.
